# Central Hyperthermia Treated with Bromocriptine

**DOI:** 10.1155/2017/1712083

**Published:** 2017-02-28

**Authors:** P. Natteru, P. George, R. Bell, P. Nattanmai, C. R. Newey

**Affiliations:** ^1^Department of Neurology, University of Missouri, 5 Hospital Drive, CE 540, Columbia, MO 65211, USA; ^2^Department of Neurology, Cerebrovascular Center, Cleveland Clinic, 9500 Euclid Avenue, Cleveland, OH 44125, USA; ^3^Department of Surgery, Division of Neurosurgery, University of Missouri, 1 Hospital Drive, Columbia, MO 65211, USA

## Abstract

*Introduction*. Central hyperthermia is common in patients with brain injury. It typically has a rapid onset with high temperatures and marked fluctuations and responds poorly to antibiotics and antipyretics. It is also associated with worse outcomes in the brain injured patient. Recognizing this, it is important to aggressively manage it.* Case Report*. We report a 34-year-old male with a right thalamic hemorrhage extending to the midbrain and into the ventricles. During his admission, he developed intractable fevers with core temperatures as high as 39.3°C. Infectious workup was unremarkable. The fever persisted despite empiric antibiotics, antipyretics, and cooling wraps. Bromocriptine was started resulting in control of the central hyperthermia. The fever spikes were reduced to minor fluctuations that significantly worsened with any attempt to wean off the bromocriptine.* Conclusion*. Diagnosing and managing central hyperthermia can be challenging. The use of bromocriptine can be beneficial as we have reported.

## 1. Introduction

In general, hyperthermia is considered secondary to an infectious etiology [1, 2]. However, in neurologically injured patients, hyperthermia may be related to the underlying brain injury and is associated with worse outcomes [3, 4]. Central hyperthermia has a rapid onset of temperature with marked fluctuations [5]. It may be a result of damage or dysfunction to central fever control centers, such as at the level of the diencephalon [6, 7]. This region regulates core temperatures [6, 7]. Any damage to this area can disrupt the thermoregulatory apparatus of the body, as evidenced in about 42% of autopsies in brain injured patients [6, 7]. Theories have been suggested to explain the role of the hypothalamus in central hyperthermia [6–8]. It may be the selective loss of warm-sensitive neurons, the osmotic changes detected by the organum vasculosum laminae terminalis (OVLT), or the humoral changes (progesterone, prostaglandin) modifying the firing rate of heat sensitive neurons in the medial preoptic nucleus (MPO) [8].

There has been increasing evidence to show that central hyperthermia is a poor responder to antipyretics [9]. Thus, it may require a multimodal approach to management that includes additional medications such as bromocriptine and/or surface or intravascular cooling device [10].

In this paper, we present a 34-year-old male with prolonged central fever after intracerebral hemorrhage of the thalamus and midbrain. The prolonged fever was controlled with the administration of bromocriptine which we graphically represent along with a review of the literature.

## 2. Case

**Figure 1:**
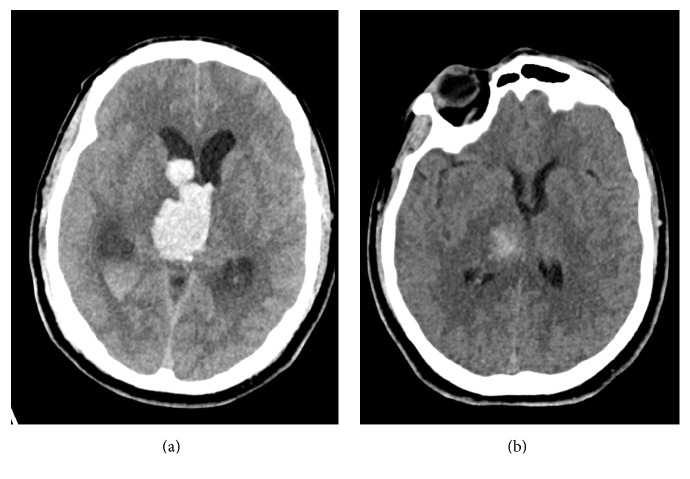
Computed tomography (CT) of the head. (a) CT head on admission showing a right thalamic hemorrhage with intraventricular extension. Note the obstructive hydrocephalus. (b) CT head on hospital day 9 showing evolution of the right thalamic hemorrhage and resolution of the obstructive hydrocephalus with external ventricular drainage (not shown) and after intraventricular tissue plasminogen activator (tPA).

A 34-year-old Hispanic male was found by his family unresponsive to verbal and noxious stimulation. His arms were asymmetrically extended. Emergency medical service was called. He was intubated in the field for airway protection and transported to the emergency department. His initial Glasgow Coma Scale (GCS) score was 5 (eyes: 1, verbal: 1, motor: 3). His computed tomography (CT) scan of the head showed a right thalamic hemorrhage (10.4 cc) with extension into the upper midbrain along with intraventricular extension resulting in obstructive hydrocephalus (Figure 1(a)).

The initial blood pressure (BP) was 163/115 mmHg. He was started on a nicardipine infusion to achieve systolic BP of <140 mmHg. His right pupil was dilated (4 mm) and nonreactive. He had extensor posturing of all extremities. An emergent external ventricular drain (EVD) was placed. Cerebral angiography was performed to evaluate for underlying aneurysm, vertebral dissection, arteriovenous malformation, or dural arteriovenous fistula. The cerebral angiography was negative. Magnetic resonance imaging (MRI) confirmed the hemorrhage without identifying any additional abnormality. Electroencephalogram (EEG) was negative for seizures. He was subsequently treated with intraventricular tissue plasminogen activator (t-PA; 1 mg q8 h) for three days (total 9 doses). The intraventricular hemorrhage improved significantly by day 9 of hospitalization (Figure 1(b)). His neurological examination, however, remained poor. EVD weaning was attempted. After multiple failed attempts, a ventriculoperitoneal shunt was placed.

During his admission, his core temperature rose to 39.3°C (102.7°F). Acetaminophen and cooling wraps (Gaymar Medi-Therm Hyper/Hypothermia System, Stryker, Kalamazoo, MI, USA) were initiated to control the fever. He was also on a fentanyl infusion for light sedation. A complete fever workup only showed leukocytosis. He was treated for presumed aspiration pneumonia. His fevers persisted with marked fluctuations despite antibiotic therapy. Other differential diagnoses such as malignant hyperthermia and neuroleptic malignant syndrome were ruled out due to normal creatinine kinase level (81 units/L; normal 20–200 units/L) and lack of causative medication.

**Figure 2:**
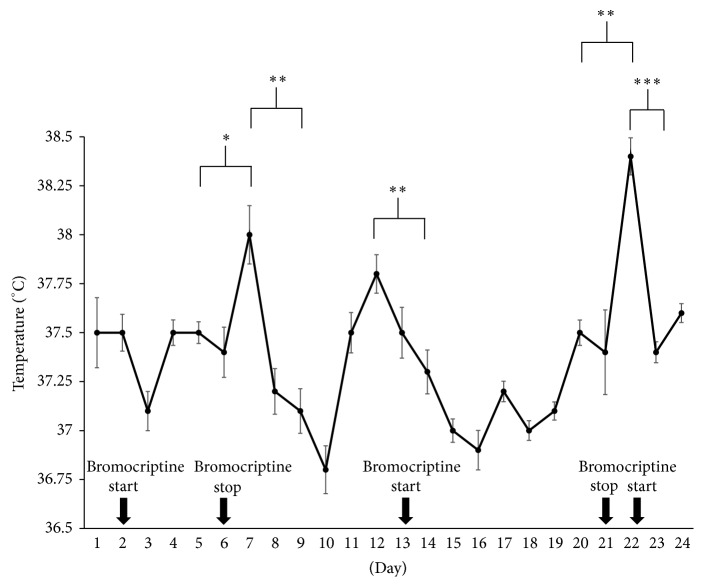
Temperature graph. Average core temperature for each day during hospital course is graphically shown. Error bars are standard errors of the mean. Bromocriptine was started on days 2, 13, and 22. It was stopped on days 6 and 21. Immediately after discontinuation of bromocriptine, there was a significant increase in core temperature. After resuming bromocriptine, there was a significant decrease in core temperature. ^*∗*^*p* < 0.05, ^*∗∗*^*p* < 0.001, and ^*∗∗∗*^*p* < 0.0001.

He was started on bromocriptine in addition to the antipyretics and cooling wraps for treatment of his central hyperthermia. The fever spikes reduced to minor fluctuations that worsened with each attempt to wean off the bromocriptine (Figure 2). Cultures remained negative. Figure 2 is a graph of the average core temperature (+/− standard error of the mean) and association of bromocriptine initiation and discontinuation. Note the significant improvement with initiation of bromocriptine and the significant worsening of fever with discontinuation of bromocriptine. There was no correlation with the resolution of the intracerebral and intraventricular hemorrhages and the administration of intraventricular tPA with the fever. Statistical analysis was performed using analysis of variance (ANOVA) on GraphPad Prism 7 (La Jolla, CA, USA). A *p* value of <0.05 was considered significant.

He required a tracheostomy and percutaneous gastrostomy tube. Neurologically, he remained in a coma with asymmetric posturing of the upper extremities and extension of the lower extremities. He was eventually transferred to a long term acute care facility.

## 3. Discussion

This case highlights the effectiveness of bromocriptine to treat fever of central origin. Neurogenic fever can occur alone or in conjunction with other autonomic (e.g., tachycardia, tachypnea, diaphoresis, and pupillary changes) and motor findings (e.g., extensor posturing) [11]. These are collectively termed paroxysmal sympathetic hyperactivity (PSH).

Hyperthermia of central origin has a rapid onset, high temperature, marked fluctuation, and poor to no response to antipyretics [9]. Fever is an independent variable in patients with neurologic injury and usually suggests worse outcomes [12]. In a retrospective study of 74 patients in the neurosciences intensive care unit with poststroke central hyperthermia, nearly 70% of the patients with fever expired within a month, especially those with temperatures > 39°C [5].

The features of central hyperthermia are hypothesized to be due to the compression of hypothalamic and brain stem thermoregulatory centers [13]. Several physiological pathways contribute to the central thermoregulation and are coordinated by the hypothalamus [14]. These areas include the lateral parabrachial nucleus at the junction of the pons and medulla, inputs from spinothalamocortical relay pathways, and the preoptic area (POA) of the hypothalamus [15]. The median preoptic nucleus within the POA is temperature sensitive, especially to heat [14]. In addition to the hypothalamus, the brainstem also plays a significant role in central thermoregulation through brown adipose thermogenesis (BAT) [10]. The rostral ventromedial medulla augments BAT, whereas the ventrolateral medulla (nucleus of the solitary tract) and the ventromedial midbrain (periaqueductal gray) inhibit BAT [16, 17]. These anatomical considerations are the reason why injury to the brainstem significantly alters the homeostasis and can lead to central hyperthermia, as seen in 64% of patients [5]. We suspect that disruption of the hypothalamus and/or ventromedial midbrain resulted in the central fever in our patient given the location of his hemorrhages.

Criteria for diagnosing central hyperthermia have been suggested: (a) high fever with temperatures > 39°C within 24 hours after onset of stroke, (b) no prior infections or fevers at least 1 week prior to onset of stroke, and (c) negative workup for fever of infectious origin [18]. Our patient met all the criteria for diagnosis of central hyperthermia.

Treatment of central hyperthermia typically requires a multimodal approach. Options include medications like bromocriptine or baclofen with or without surface or intravascular cooling devices [9, 19–22]. In our patient, we added bromocriptine to surface cooling and antipyretic to treat the central fever. In addition to bromocriptine, the patient did receive fentanyl infusion for mild sedation to allow for frequent neurological assessments.

Bromocriptine is a dopamine D2 agonist that acts on the corpus striatum and the hypothalamus [23]. It has been known to help in PSH, because of its action on dopaminergic transmission [24, 25]. Unfortunately, there is no systematic study on the efficacy and safety of dopamine agonists in traumatic brain injury [26]. Much of the current literature are case reports and small studies with inherent bias [26]. It may be best to individualize the therapy for each patient. In clinical practice, the use of bromocriptine has been less robust for the treatment of the constellation of findings associated with PSH except for central hyperthermia [11].

Other treatment options for central hyperthermia include baclofen, a GABA agonist, which acts on the raphe nuclei and inhibits BAT which in turn can suppress the core body temperature [21]. Baclofen use in the neurocritical care unit, however, is limited by side effects such as drowsiness, tiredness, and muscle weakness of the affected or unaffected limbs [27]. Lastly cooling devices are effective in reaching normothermia [28]. However, these devices have risks such as infection and thrombosis with intravascular cooling and skin breakdown with the cooling wraps [29]. Both increase risk of shivering [28, 30].

## 4. Conclusion

Our case report illustrates that bromocriptine is an effective treatment option for central hyperthermia. Future studies should evaluate improvement in temperature management and outcomes in patients treated with bromocriptine in the setting of neurogenic fever.
